# Bridge from central extracorporeal life support is a risk factor of cerebrovascular accidents after durable left ventricular assist device implantation

**DOI:** 10.1007/s10047-021-01303-2

**Published:** 2021-12-06

**Authors:** Kohei Tonai, Satsuki Fukushima, Naoki Tadokoro, Satoshi Kainuma, Naonori Kawamoto, Takashi Kakuta, Ayumi Koga-Ikuta, Takuya Watanabe, Osamu Seguchi, Yasumasa Tsukamoto, Norihide Fukushima, Tomoyuki Fujita

**Affiliations:** 1 Department of Cardiac Surgery, National Cerebral and Cardiovascular Center, 6-1 Kishibeshimmachi, Suita, Osaka 564-8565 Japan; 2 Department of Transplant Medicine, National Cerebral and Cardiovascular Center, Suita, Osaka Japan

**Keywords:** Heart failure, Ventricular assist device, Extracorporeal life support, Bridge-to-bridge, Stroke

## Abstract

A bridging strategy from extracorporeal life support (ECLS) is effective in salvage and a bridge to recovery or to a durable left ventricular assist device (LVAD) for acute refractory heart failure. However, the correlation of this strategy with adverse events after durable LVAD implantation has not been fully investigated. This study enrolled 158 consecutive patients who had either the HeartMate II or HeartMate 3 and were implanted for bridge-to-transplantation. These devices were implanted as the primary mechanical support device in 115 patients, whereas the remaining 43 underwent LVAD implantation as the bridge from central ECLS. The primary study endpoint was all-cause mortality and cerebrovascular accidents (CVAs) after durable LVAD implantation, and the secondary endpoints were adverse events. Overall survival was not significantly different between the two groups. In contrast, the probability of CVAs was significantly greater in the bridge group than in the primary group (probability of CVAs, *P* = 0.002; log-rank test). In Cox multivariate logistic regression analysis, a bridge from central ECLS was an independent predictive factor of CVAs (hazard ratio 4.27, 95% confidence interval 1.43–12.8; *P* = 0.0095). Patients who are bridged from central ECLS are more frequently complicated by CVAs compared with those who undergo primary implantation of a durable LVAD, but survival is not significantly different between the two groups. A bridge from central ECLS is an independent predictive factor of CVAs post-implantation of an LVAD.

## Introduction

Extracorporeal life support (ECLS) is initiated in patients with acute refractory congestive heart failure as the initial mechanical circulatory support. Patients who show sufficient functional recovery under ECLS can wean from mechanical support, whereas those failing to show functional recovery require upgrade of mechanical support to a durable left ventricular assist device (LVAD) [1, 2]. We recently reported the feasibility, safety, and therapeutic efficacy of central ECLS on salvage and/or bridge to recovery or to a durable LVAD for acute refractory congestive heart failure caused by myocardial infarction, myocarditis, or cardiomyopathy [3]. However, this bridge from central ECLS to a durable LVAD strategy is associated with several positive and negative factors [4–6].

In the bridge strategy, hemodynamic stabilization and left ventricular unloading are completed under central ECLS before durable LVAD implantation. Therefore, hemodynamic and structural changes by durable LVAD implantation are minimal, potentially enhancing recovery from this implant surgery [6]. In contrast, insufficient general recovery from ECLS surgery at the time of durable LVAD implant surgery may be associated with postoperative complications, such as poor morbidity and/or infectious diseases that prolong the in-hospital stay. Additionally, intrapericardial adhesion induced by central ECLS surgery may potentiate blood product use and exacerbate coagulopathic change, which lead to a suboptimal anticoagulation state after durable LVAD implantation. Our research group recently reported that this bridge strategy was more strongly associated with 1-year composite events compared with primary durable LVAD implantation [3]. Imamura et al. also recently reported that this bridge strategy was associated with lower 3-year mortality compared with primary durable LVAD implantation [7]. However, the underlying mechanisms that determine the difference between primary and bridge implantation are not fully understood.

We hypothesized that adverse events associated with the bridge strategy might be predicted by background/characteristics or intraoperative variables at durable LVAD implantation surgery. To test this hypothesis, we aimed to review in-hospital and mid-term outcomes of a bridge from central ECLS to the durable LVAD strategy compared with those of primary LVAD implant surgery, and to examine predictive factors associated with adverse events after durable LVAD implantation.

## Materials and methods

### Study cohort and data collection

The institutional surgical database contained information on a consecutive series of 158 patients who underwent either the HeartMate II (Abbott, Chicago, IL, USA) or HeartMate 3 (Abbott) device for bridge-to-transplantation in the Department of Cardiac Surgery between April 2013 and September 2020. The patients’ medical charts, surgical reports, and referral letters were reviewed to collect study data. Clinical follow-up was completed at the end of the study in all patients. Data collection was performed in November 2020. Either patients or their legal representatives provided written informed consent for surgery and the use of their data for diagnostic and research purposes preoperatively. The study was conducted in compliance with the Declaration of Helsinki and the International Conference on Harmonization/Good Clinical Practice. The study was approved by the National Cerebral and Cardiovascular Center Institutional Review Board for Clinical Research (approval number: M30-026).

### Study endpoints

The primary study endpoint was all-cause mortality and cerebrovascular accidents (CVAs) after durable LVAD implantation, and the secondary endpoints were adverse events defined in the Interagency Registry for Mechanically Assisted Circulatory Support report [8], apart from CVAs. CVAs consisted of any stroke event, transient ischemic attack, or seizures. Stroke was further categorized by subtype (ischemic and hemorrhagic) and by severity with the modified Rankin Scale (mRS). The mRS was divided into the following severity classifications: nondisabling (mRS score 0–3) or disabling (mRS score ≥ 4) strokes. Additionally, driveline infection was defined as a positive culture from the skin and/or tissue surrounding the driveline, coupled with the requirement to treat with antimicrobial therapy when there was clinical evidence of infection, such as pain, fever, drainage, or leukocytosis [9]. Pump failure was defined as cessation of any component of the device system to operate to its designed performance specifications or otherwise failure to perform as intended [10]. Re-sternotomy was performed in patients with sustained chest drain output, pericardial tamponade, or deep sternal wound infection.

### Indication and selection of a durable LVAD

All patients were pathologically diagnosed on the basis of biopsy specimens of the right ventricle (RV) before LVAD implantation. LVAD was implanted as bridge-to-transplantation in 157 patients who were listed in the Organ Transplantation Network, Japan before surgery, whereas one patient underwent durable LVAD implantation as the destination therapy. The primary selection of the durable LVAD device was the HeartMate II (*n* = 120) between April 2013 and January 2019 and the HeartMate3 (*n* = 38) between August 2019 and September 2020.

### Cohort grouping and surgical procedure

A durable LVAD was implanted as the primary mechanical support device in 115 patients (primary group), whereas the remaining 43 underwent durable LVAD implantation as the bridge from central ECLS (bridge group). In the bridge group, the mean duration of central ECLS was 52.4 ± 44.4 days (range 3–173 days). Initial central ECLS was an extracorporeal LVAD in 30 (69.8%) patients, extracorporeal bilateral ventricular assist device in 7 (16.3%), and extracorporeal membrane oxygenation by central cannulations in 6 (1%).

The durable LVAD was implanted by the median sternotomy approach in all patients. In the bridge group, the left ventricular apex cuff of the central ECLS was completely removed to replace the cuff of the HeartMate II or HeartMate 3. Tricuspid annuloplasty was performed by using a prosthetic ring in patients with moderate or more tricuspid regurgitation preoperatively (*n* = 45). Aortic valve plasty was performed by the Park’s stich [11] in patients with mild or more aortic valve regurgitation preoperatively (*n* = 19). In patients who failed to wean off cardiopulmonary bypass under durable LVAD support owing to poor RV function, mechanical RV support was added by cannulations into the main pulmonary artery and right atrium via the femoral vein, which was connected to an extracorporeal membrane oxygenation circuit (*n* = 4).

### Perioperative medical treatments and laboratory examinations

In patients who were on a vitamin K antagonist before surgery, prothrombin complex was administered before the skin incision to a target international normalized ratio (INR) of < 1.5. The chest was not left open in any patients at entry to the intensive care unit. Considering that early postoperative heparinization reduces hemolysis [12], heparin infusion was started within 24–48 h after implantation, depending upon the chest drain output. This infusion was titrated by targeting an activated prothrombin time of 50–70 s until the INR reached 2.0. A vitamin K antagonist was started at days 1–2 postoperatively by targeting an INR of 2.0–3.0. Aspirin (100 mg daily) was started when the platelet count in the blood was > 100,000/mm^3^.

All patients were examined by standard transthoracic echocardiography and a right heart catheter study within 1 month preoperatively and postoperatively. A blood test was performed 1 day before surgery and daily for 1 week postoperatively.

### Statistical analysis

All statistical analysis was performed by using JMP software, ver. 15.2 (SAS Institute Inc., Cary, NC, USA). For comparison of preoperative background and characteristics of patients between the primary and bridge groups, continuous variables were compared by using the unpaired *t* test and categorical variables were compared using the chi-square test. Continuous variables are shown as mean ± standard error and categorical variables are shown as numbers and percentages. Overall survival and CVA-free survival were estimated using Kaplan–Meier curves and compared across groups using the log-rank test. Multivariable logistic regression models were used to identify the risk factors for CVAs during the follow-up period. Statistical significance was defined as a *P* value < 0.05.

## Results

### Patients’ characteristics and cardiovascular parameters

For the background characteristics of the patients, idiopathic dilated cardiomyopathy was the major etiology in the primary group, whereas ischemic cardiomyopathy and myocarditis were the major etiologies in the bridge group (Table 1). With regard to cardiovascular characteristics, the size of the left ventricle (LV) was significantly smaller in the bridge group than in the primary group (Table 2). Additionally, pulmonary artery pressure (PAP) and pulmonary capillary wedge pressure (PCWP) were significantly lower in the bridge group than in the primary group. Serum albumin levels were significantly lower in the bridge group than in the primary group, whereas kidney function was more preserved in the bridge group than in the primary group.

**Table 1:** Comparison of baseline clinical characteristics on admission between the primary and bridge groups

	Primary	Bridge	*P* value
	(*n* = 115)	(*n* = 43)	
Age (years)	45.4 ± 1.1	44.7 ± 1.9	0.75
Male, *n* (%)	82 (71%)	29 (67%)	0.64
Body mass index (kg/m^2^)	21.0 ± 0.4	22.3 ± 0.6	0.066
Body surface area (m^2^)	1.6 ± 0.02	1.7 ± 0.03	0.16
VAD			
HeartMate II, *n* (%)	88 (77%)	32 (74%)	0.78
HeartMate 3, *n* (%)	27 (23%)	11 (26%)	
INTERMACS profile level on admission			< 0.0001
1	26 (23%)	42 (98%)	
2	88 (76%)	1 (2%)	
3	1 (1%)	0 (0%)	
Etiology			
DCM	71 (61%)	15 (35%)	< 0.0001
dHCM	13 (11%)	5 (12%)	
ICM	10 (9%)	12 (28%)	
Myocarditis	3 (3%)	7 (16%)	
Others	18 (16%)	3 (7%)	
Medical history			
Atrial fibrillation	6 (21%)	0 (0%)	0.036
Diabetes	36 (31%)	10 (23%)	0.43
Stroke or TIA	0 (0%)	0 (0%)	
Cancer	1 (1%)	0 (0%)	0.42
Hypertension	10 (9%)	9 (21%)	0.045
Known PFO	4 (3%)	3 (7%)	0.36
Coronary artery bypass	5 (4%)	2 (5%)	0.93
Valve replacement/repair	6 (5%)	2 (5%)	0.88
Dyslipidemia	49 (43%)	14 (33%)	0.25
Smoker	60 (52%)	19 (44%)	0.37

*VAD* ventricular assist device, *INTERMACS* Interagency Registry for Mechanically Assisted Circulatory Support, *DCM* dilated cardiomyopathy, *ICM* ischemic cardiomyopathy, *dHCM* dilated phase of hypertrophic cardiomyopathy, *TIA* transient ischemic attack, *PFO* patent foramen ovale

**Table 2:** Comparison of preoperative parameters before durable LVAD implantation surgery between the primary and bridge groups

	Primary	Bridge	*P* value
	(*n* = 115)	(*n* = 43)	
Left ventricular end-diastolic diameter (mm)	71.5 ± 1.2	56.8 ± 2.0	< 0.0001
Left ventricular end-systolic diameter (mm)	65.1 ± 1.3	51.7 ± 2.0	< 0.0001
Left ventricular ejection fraction	19.5 ± 0.8	15.8 ± 1.3	0.0017
Arterial blood pressure (mmHg)			
Systolic	88.5 ± 1.2	88.5 ± 2.3	0.998
Diastolic	59.0 ± 1.1	62.9 ± 2.2	0.115
Mean	65.2 ± 2.2	51.9 ± 3.7	0.0026
Cardiac index (l/min/m^2^)	1.9 ± 0.05	2.7 ± 0.09	< 0.0001
Pulmonary artery pressure (mmHg)			
Systolic	41.2 ± 1.6	30.2 ± 2.9	0.0009
Diastolic	21.2 ± 1.0	15.5 ± 1.8	0.0075
Mean	29.0 ± 1.1	19.3 ± 2.0	< 0.0001
Pulmonary capillary wedge pressure (mmHg)	20.5 ± 0.9	11.0 ± 1.6	< 0.0001
Pulmonary vascular resistance, Wood units	2.9 ± 0.2	2.0 ± 0.3	0.0093
Right atrial pressure (mmHg)	8.0 ± 0.7	8.0 ± 1.2	0.955
Right ventricular stroke work index (g/m/beat/m^2^)	2.6 ± 0.2	4.1 ± 0.4	0.0004
Preoperative laboratory data			
TP (g/dl)	6.6 ± 0.07	6.0 ± 0.1	< 0.0001
Alb (g/dl)	3.9 ± 0.05	3.2 ± 0.08	< 0.0001
AST (IU/l)	50.1 ± 17.7	31.5 ± 28.9	0.584
ALT (IU/l)	42.9 ± 15.2	22.9 ± 24.8	0.492
T-Bil (mg/dl)	1.0 ± 0.09	1.1 ± 0.14	0.552
BUN (pg/ml)	18.8 ± 0.9	13.0 ± 1.4	0.0006
Cre (mg/dl)	1.1 ± 0.03	0.8 ± 0.05	< 0.0001
LDH (U/l)	263.2 ± 25.1	444.9 ± 40.9	0.0002

*LVAD* left ventricular assist device, *TP* total protein, *Alb* albumin, *AST* aspartate transferase, *ALT* alanine transferase, *T-Bil* total bilirubin, *BUN* blood urea nitrogen, *Cre* creatinine, *LDH* lactate dehydrogenase

### Procedural and in-hospital outcomes

The operation time was significantly longer with greater use of blood products in the bridge group compared with the primary group (Table 3). However, the timing of starting heparin infusion was not significantly different between the two groups.

**Table 3:** Comparison of perioperative variables and postoperative outcomes between the primary and bridge groups

	Primary	Bridge	*P* value
	(*n* = 115)	(*n* = 43)	
Operation			
Time (min)	274.8 ± 10.6	361.2 ± 17.3	< 0.0001
Transfusion: RBC, units	12.9 ± 1.0	20.9 ± 1.7	< 0.0001
Transfusion: FFP, units	19.6 ± 1.7	28.9 ± 2.9	0.0066
Transfusion: Platelets, units	31.1 ± 1.6	41.9 ± 2.6	0.0005
Postoperative intubation (h)	14.6 ± 1.8	16.7 ± 3.0	0.54
Postoperative duration until heparin initiation (h)	30.4 ± 2.1	26.4 ± 3.3	0.32
ICU stay (days)	4.4 ± 0.4	4.9 ± 0.7	0.52
Hospital stay (days)	126.3 ± 8.7	109.1 ± 15.7	0.34
Postoperative RVAD, *n* (%)	3 (2.6)	1 (2.3)	0.92
Catheter parameters at 1 month			
Cardiac Index (l/min/m^2^)	2.7 ± 0.05	2.8 ± 0.09	0.38
Pulmonary capillary wedge pressure (mmHg)	5.4 ± 0.4	6.2 ± 0.6	0.22
Mean pulmonary artery pressure (mmHg)	14.2 ± 0.4	14.5 ± 0.6	0.69
Right atrial pressure (mmHg)	5.4 ± 0.4	6.2 ± 0.6	0.26
Right ventricular stroke work index (g/m/beat/m^2^)	4.1 ± 0.2	4.3 ± 0.3	0.58
Early events (until discharge)			
Cerebrovascular accidents (%)	6 (6.8)	8 (29.6)	0.0034
Drive line infection (%)	2 (2.3)	0 (0)	0.3
Pump failure (%)	2 (2.3)	2 (7.4)	0.24
Re-sternotomy (%)	21 (18.3)	10 (24.4)	0.41

*RBC* red blood cell, *FFP* fresh-frozen plasma, *ICU* intensive care unit, *RVAD* right ventricular assist device

In-hospital or 30-day mortality occurred in two patients in the primary group and in one patient in the bridge group. The cause of death was sepsis in two patients and pneumonia in one patient.

Postoperative recovery was not significantly different between the two groups, except for CVAs, which occurred significantly more frequently in the bridge group than in the primary group. Postoperative transesophageal echocardiography, which was performed at 32 ± 8 days postoperatively, showed no significant difference in LV size or ejection fraction between the groups. A right heart catheter study, which was performed at 54 ± 23 days postoperatively, showed that the cardiac index, PAP, and PCWP were not significantly different between the two groups.

### Mid-term CVAs in the bridge group

**Fig. 1:**
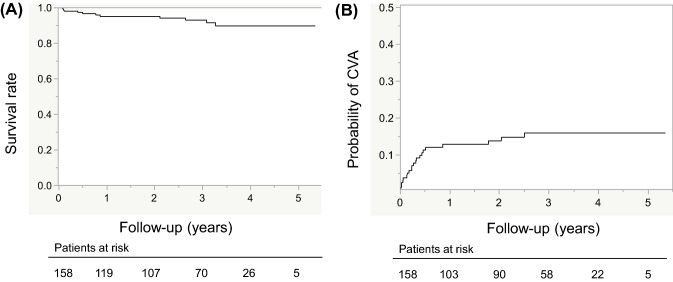
(**A**) Long-term survival of the total cohort. Kaplan–Meier curves show 1- to 5-year rates of survival. (**B**) Comparison of the cumulative incidence of CVAs in the total cohort. Kaplan–Meier curves show 1- to 5-year rates of incidence. *CVAs* cerebrovascular accidents

**Fig. 2:**
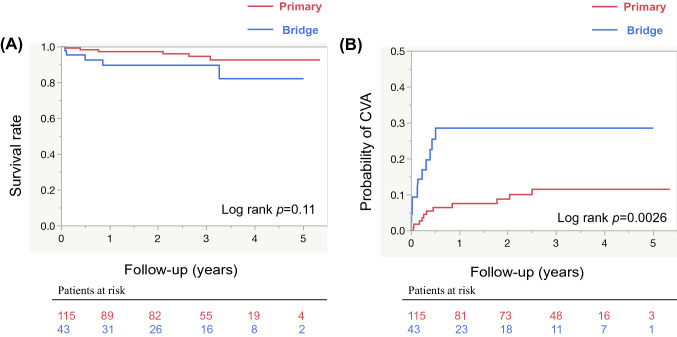
(**A**) Comparison of long-term survival between the bridge group and the primary group. Kaplan–Meier curves show 1- to 5-year rates of survival. (**B**) Comparison of the cumulative incidence of CVAs between the two groups. Kaplan–Meier curves show 1- to 5-year rates of incidence. The blue and red lines show the survival curve of the bridge and the primary groups, respectively. *CVAs* cerebrovascular accidents

Survival of the total cohort was 95% at 1 year and 93% at 3 years (Fig. 1A), whereas the probability of CVAs was 14% at 1 year and 16% at 3 years (Fig. 1B). Overall survival was not significantly different between the two groups (Fig. 2A). Survival at 1 year was 97% in the primary group versus 90% in the bridge group and that at 3 years was 94% in the primary group versus 90% in the bridge group. An orthotropic heart transplant was performed in 31 (27%) patients in the primary group and in 5 (11.6%) patients in the bridge group (*P* = 0.032).

**Fig. 3:**
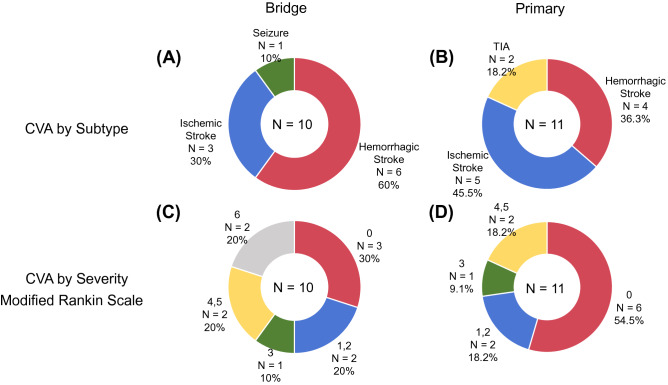
(**A**) Distribution of subtypes of CVAs in the bridge group. (**B**) Distribution of subtypes of CVAs in the primary group. (**C**) Distribution of the mRS severity score of CVAs in the bridge group. (**D**) Distribution of the mRS severity score of CVAs in the primary group. *CVAs* cerebrovascular accidents, *mRS* modified Rankin Scale

The probability of CVAs was significantly greater in the bridge group compared with the primary group (Fig. 2B). Until the last follow-up, 11 (9.6%) patients in the primary group and 10 (25.6%) patients in the bridge group were complicated by CVAs. Notably, all CVAs occurred within 1 year postoperatively in the bridge group. With regard to the type of CVA, hemorrhagic events predominated in the bridge group and ischemic events predominated in the primary group (Fig. 3). Moreover, disabling CVAs predominated in the bridge group, whereas non-disabling stroke predominated in the primary group.

### Predictive factors of CVAs after durable LVAD implantation

Predictive factors of CVAs post-durable LVAD implantation were examined. A total of 158 patients were included in this analysis. In Cox univariate logistic regression analysis, delayed heparinization (> 24 h), a preoperative small LV end-systolic diameter (< 50 mm), long operation time (> 300 min), and a bridge from central ECLS were significant factors that predicted CVAs post-durable LVAD implantation (Table 4). In Cox multivariate logistic regression analysis, a bridge from central ECLS was the only independent predictive factor of CVAs with a hazard ratio of 4.27. Delayed heparinization also had a strong correlation with CVAs, though the difference was not statistically significant.

**Table 4:** Cox hazard analysis for cerebrovascular accidents in the entire cohort

Risk factors	Univariate			Multivariate		
	Hazard ratio	95% confidence interval	*P* value	Hazard ratio	95% confidence interval	*P* value
Age (> 50)	0.81	0.33–1.98	0.64	0.78	0.29–2.17	0.64
Sex (male)	0.84	0.34–2.07	0.71			
Small BSA (< 1.5 m^2^)	1.91	0.80–4.54	0.15			
Delayed heparinization (> 24 h)	2.91	1.02–8.26	0.045	2.9	0.98–8.56	0.053
Preoperative small LVDs (< 50 mm)	2.98	1.25–7.12	0.014	2.36	0.76–7.35	0.14
Preoperative inflammation (WBC > 10,000/mm^3^)	1.12	0.26–4.79	0.88			
Preoperative hypoalbuminemia (< 3.0 g/dl)	1.68	0.49–5.71	0.41			
Long operation time (> 300 min)	3.45	1.45–8.22	0.0053			
Bridging with extracorporeal VAD	3.38	1.46–7.81	0.0044	4.27	1.43–12.8	0.0095
HeartMate II implantation	4.1	0.54–30.7	0.17	3.74	0.48–29.3	0.21

*LVDs* left ventricular end-systolic diameter, *WBC* white blood cell, *VAD* ventricular assist device, *BSA* body surface area

Furthermore, all patients were divided into the two groups of with or without CVAs after durable LVAD implantation. Patients who were complicated by CVAs showed a significantly smaller preoperative LV size, smaller body surface area, a longer operation time, more platelet product use, and a longer hospital stay compared with those who were not complicated by CVAs (Table 5). Half of the patients bridged with ECLS were complicated by CVAs, whereas no patients who were bridged with a transcatheter LVAD (Impella; Abiomed Inc., Danvers, MA, USA) were complicated by CVAs.

**Table 5:** Subgroup analysis of perioperative variables and postoperative outcomes in patients with CVAs and without CVAs

	CVAs (−)	CVAs (+)	*P* value
	(*n* = 136)	(*n* = 22)	
Age (years)	45.0 ± 1.1	45.1 ± 2.6	0.99
Male, *n* (%)	96 (71)	15 (68)	0.78
Body Surface area (m^2^)	1.7 ± 0.02	1.6 ± 0.04	0.039
Preoperative LVDd	65.4 ± 1.3	61.5 ± 3.1	0.043
Preoperative LVDs	62.3 ± 1.3	55.5 ± 3.1	0.044
Bridging from Extracorporeal VAD, *n* (%)	25 (18.5)	11 (50)	0.0024
Bridging from impella, *n* (%)	8 (5.9)	0 (0)	0.12
Operative time of central ECLS surgery (min)	268.3 ± 19.4	234.7 ± 28.7	0.34
Operative time of durable VAD surgery (min)	288.5 ± 10.1	356.9 ± 25.1	0.013
Transfusion			
RBC, units	14.6 ± 1.0	17.6 ± 2.4	0.24
FFP, units	21.6 ± 1.7	26.1 ± 4.1	0.31
Platelets, units	32.7 ± 1.5	42.3 ± 3.6	0.017
Postoperative RVAD requirement	4 (3)	0	0.27
Postoperative intubation (h)	15.4 ± 1.7	13.3 ± 4.3	0.65
Postoperative duration until heparin initiation (h)	28.2 ± 1.9	37.0 ± 5.1	0.1
ICU stay (days)	4.6 ± 0.4	4.3 ± 0.9	0.82
Hospital stay (days)	111.2 ± 8.0	174.8 ± 17.5	0.0013
Drive Line Infection, *n* (%)	25 (18.5)	5 (22.7)	0.65

*CVAs* cerebrovascular accidents, *LVDd* left ventricular end-diastolic diameter, *LVDs* left ventricular end-systolic diameter, *VAD* ventricular assist device, *ECLS* extracorporeal life support, *RBC* red blood cell, *FFP* fresh-frozen plasma, *RVAD* right ventricular assist device, *ICU* intensive care unit

## Discussion

This study examined patients who were bridged from central ECLS and were more frequently complicated by CVAs compared with those who underwent primary implantation of HeartMate II or HeartMate 3. Survival outcome was not significantly different between the two groups. The postoperative survival rate and cumulative incidence of CVAs in this cohort are comparable with the outcomes of previous studies [13–17]. Notably, this bridge strategy was the only independent predictive factor of CVAs post-implantation of HeartMate II or HeartMate 3.

Various factors could be associated with our finding that a bridge from central ECLS was the only independent predictive factor of CVAs as follows. First, ischemic and myocarditis etiologies, which predominated in the bridge group, potentiate regional motion abnormalities in the LV wall and result in formation of LV thrombus. However, idiopathic dilated cardiomyopathy, which predominated in the primary group, causes a reduction in LV wall motion globally and homogeneously. Second, LV size at the durable LVAD implantation was significantly smaller in the bridge group compared with the primary group. A smaller LV under durable LVAD is more likely to lead to formation of wedge thrombus [18]. Third, a poor nutritional state at durable LVAD implantation, represented by serum albumin levels, in the bridge group may have been associated with CVAs post-durable LVAD implantation [19]. Fourth, refractory cardiogenic shock before admission causes severe multiorgan dysfunction, which might not have fully recovered before durable LVAD implantation. Finally, a prolonged operation time with predominant use of blood products in the bridge group could have been the cause of CVAs. Adhesiolysis of the pericardial space was additionally required before durable LVAD implantation in all patients in the bridge group, whereas only 20 patients required intrapericardial adhesiolysis owing to previous cardiac procedures in the primary group. Complicated and long operation requiring a large amount of blood products could result in postoperative delayed heparinization, which showed a strong tendency to evoke CVAs. Our institute recently showed that hemolysis in HeartMate II was correlated with delayed heparinization. Thus, we consider that optimum coagulation profile should be established as soon as the surgery is completed to prevent thrombogenic complications, such as CVAs or hemolysis.

The finding that CVAs in the bridge strategy could be caused by etiology, LV size, nutritional state, or complicated surgery can be explained by the timing and severity of CVAs in our study. All CVAs in the bridge group occurred within 1 year after LVAD implantation. Additionally, hemorrhagic and disabling stroke predominated in the bridge group, representing a coagulopathic state potentially associated with a poor nutritional state. However, once the general condition became stabilized under durable LVAD support, outcomes were not different between the bridge and primary groups. Therefore, physicians need to pay attention to these factors for patients under the bridge strategy. This includes prompt establishment of anticoagulant therapy for patients with regional LV wall motion abnormalities and/or a small LV size, a durable LVAD implant under a normalized nutritional state, or a meticulous adhesiolysis procedure.

This study may have a limitation of its design in which durable LVADs, apart from HeartMate II or HeartMate 3, were not included. A variety of durable LVADs have been implanted in our institute depending on the feasibility or availability of the devices in the last 20 years. Among them, HeartMate II and HeartMate 3 were the most safely used primary choice of devices throughout the study period. Inclusion of other devices in this study would have substantially increased the bias in surgical indications, the implantation procedure, or the postoperative management. Additionally, our study may have been limited by the treatment strategy in Japan, in which a durable LVAD is implanted for patients who are listed as transplant candidates before surgery. Therefore, ECLS is established first for patients with acute refractory heart failure to assess candidacy for transplant followed by durable LVAD implantation. However, this bridge to candidacy strategy is practiced worldwide. Additionally, mechanisms underlying CVAs in the bridge strategy, which were described in this study, could have important implications in use of durable LVADs. As a statistical limitation, in this cohort, CVAs occurred only in 22 cases, limiting up to 4 or 5 factors which can be included in the multivariate analysis. We selected factors that were included in the multivariate analysis, based on *P* values in the univariate analysis and on cofounding tendency among the factors.

In conclusion, a bridge from central ECLS was a risk factor of CVAs after durable LVAD implantation, and these CVAs could have been associated with a small LV size, poor nutritional state, or intraoperative blood product use. Attention to these factors would improve outcomes of patients who have a bridge from central ECLS.

## Abbreviations

ECLS: Extracorporeal life support
LVAD: Left ventricular assist device
CVAs: Cerebrovascular accidents
mRS: Modified Rankin Scale
RV: Right ventricle
LV: Left ventricle
